# Cartridge-based sequencing for bedaquiline resistance detection from sputum

**DOI:** 10.5588/ijtldopen.24.0124

**Published:** 2024-09-01

**Authors:** J.D. Limberis, R.J. Nagel, D. Block, R.E. Colman, A. Nalyvayko, Z. Howard, S. Dewell, S. Chakravorty, J.Z. Metcalfe

**Affiliations:** ^1^ Division of Pulmonary and Critical Care Medicine, Zuckerberg San Francisco General Hospital and Trauma Centre, University of California, San Francisco, San Francisco, CA, USA;; ^2^ Cepheid, Sunnyvale, CA, USA;; ^3^ Division of Pulmonary, Critical Care, Sleep Medicine, and Physiology, University of San Diego, San Diego, CA, USA.; ^4^ Division of Experimental Medicine, University of California, San Francisco, San Francisco, CA, USA.

**Keywords:** tuberculosis, drug-resistant tuberculosis, bedaquiline, *rv0678*, *atpE*, Xpert Ultra, targeted next-generation sequencing

Dear Editor,

Drug-resistant TB (DR-TB) remains a major global public health problem.^1^ Bedaquiline (BDQ), an adenosine triphosphate synthase inhibitor with strong mycobactericidal activity, is a keystone drug utilized in 124 countries. However, emerging resistance to BDQ threatens to reverse DR-TB management gains.^2^ Conventional growth-based drug susceptibility testing (DST) requires high containment laboratories, is poorly scalable, and regularly commits patients to months of potentially ineffective treatments.^3^ Unlike commercial molecular TB tests such as Xpert^®^ MTB/RIF Ultra (Cepheid, Sunnyvale, CA, USA), which assess a small number of genetic loci, targeted next-generation sequencing (NGS) can rapidly sequence entire genes. This is necessary for a BDQ genotypic assay due to the dispersion of variants and has recently been endorsed by WHO.^4^ Sequencing directly from sputum depends on successful DNA extraction, a longstanding challenge given sputum’s high viscosity, variable bacterial load, and presence of DNA-degrading enzymes and polymerase chain reaction (PCR) inhibitors. No universally accepted DNA extraction method from sputum exists, and no method consistently captures high-quality DNA from low bacterial load inputs.^5^ Further, conventional sequencing library preparation for targeted NGS involves multiple enzymatic reactions and quality control steps within complex workflows, requiring expensive equipment (e.g., thermocyclers and PCR hoods) and skilled technicians. Thus, both processes are vulnerable to failures which collectively render sequencing costly and impractical in many settings. Here, we leverage the sample processing, cell lysis, DNA extraction, and PCR capabilities of a standard Xpert microfluidic cartridge to perform individual sequencing library preparations, removing more than 90% of the complex hands-on steps of the conventional workflow. We developed a novel hemi-nested duplex PCR that amplifies full-length BDQ resistance-conferring genes indicated in the recent WHO guidelines,^6^ *rv0678* and *atp*E*,* as overlapping fragments (i.e., one forward and two reverse primers for *rv0678 and* one forward and one reverse primer for *atp*E) and adds Illumina indexes and adapters, performed in a single reaction contained entirely within the microfluidic Xpert cartridge. Subsequent alignment and variant calling were done within a publicly available pipeline (www.DrDx.Me).^7^

We performed 300 base pair (bp), paired-end Illumina sequencing of *M. tuberculosis* H37Rv spike-in sputum from non-TB respiratory illnesses patients (Discovery Life Sciences, Huntsville, AL, USA); approximated to 1+ [20,000 colony forming units (CFU)], 2+ [200,000 CFU], and 3+ [2,000,000 CFU] AFB smear grades), and 10 clinical sputum sediments from individuals with Xpert-diagnosed rifampin-resistant TB across a range of bacterial loads (Table) as a single MiSeq run (MiSeq Reagent Kit v3). Phenotypic BDQ DST were available for five of ten samples from paired culture isolates. We utilized the in-cartridge hemi-nested PCR approach to produce *M. tuberculosis*-specific sequence data (12,457,090 reads, 92% passing the quality filter). For the spiked sputum samples, alignment to reference amplicons was 80% (±13%), 31% (±19%), 42% (±16%) for the AFB sputum smear 3+, 2+, and 1+ samples, respectively. We successfully detected full-length *rv0678* and *atp*E genes in all samples, with sufficient coverage (>1,000x) and quality to call variants at all positions for *atp*E, *rv0678,* and *rv0678* promoter in most samples; low coverage occurred with two samples (one AFB 3+ and one AFB 1+ spiked sample) for both genes. As expected for *M. tuberculosis* H37Rv, we detected no mutations in either gene. For the ten DR-TB clinical sputa, we successfully detected full-length *rv0678* and *atp*E genes with sufficient coverage (>1,000x) and quality to call variants at all positions in all samples (Supplementary Data Figure S1), with a median alignment to reference amplicons of 43% (interquartile range 25–75%). We determined that all samples had wild-type *atp*E. Nine clinical samples had wild-type *rv0678* sequences (with a lineage-associated *rv0678* -11C>A mutation in one sample), while the single isolate with known phenotypic BDQ resistance had a ∼60 bp duplication.^8^ This variant would not be detected by targeted probe-based assays or assays exclusively targeting the gene's coding region.

**Table 1.** Clinical sputum sediments sequenced entirely within a microfluidic cartridge in this study.*

ID	Reads	Aligned	Smear	Rifampicin	Isoniazid	Bedaquiline	*rv0678*	*atpE*
	(x10^5^)	(%)	grade				mutation	mutation
GD62	1.6	17	Negative	Resistant	Resistant	Susceptible	WT	WT
GD739	1.2	72	Negative	Resistant^†^	Resistant^†^	Susceptible	WT	WT
GD124	1.7	19	1+	Resistant	Resistant	—	WT	WT
GD518	2.2	76	1+	Resistant	Resistant	—	WT	WT
GD578	1.5	25	2+	Resistant	Resistant	Susceptible	WT	WT
GD236	2.4	27	2+	Resistant	Resistant	Susceptible	WT	WT
GD27	2.0	25	2+	Resistant	Resistant	—	WT	WT
GD532	2.5	99	3+	Resistant	Resistant	—	WT	WT
GD576	7.4	59	3+	Resistant	Resistant	—	−11C>A	WT
GD840	6.4	88	3+	Resistant	Resistant	Resistant	Structural variant	WT

* Clinical, DST, and sequencing parameters (sequencing reads and alignment with the WT reference) for clinical sputum sediments utilized in this study. DST for rifampicin and isoniazid was done using Hain line-probe assay (GenoType MTBDR*plus*), while DST for bedaquiline was done phenotypically in the MGIT (BD, Franklin Lakes, MD, USA) system. A ∼60 bp duplication was seen in *rv0678* of GD840 (and a lineage-specific SNP upstream of *rv0678* in GD576). Low alignment rates are indicative of PCR artifacts.

† DST done on culture isolate and not directly on sputum.

— = DST not performed. WT = wild-type; DST = drug susceptibility testing.

Identifying genotypic BDQ resistance is complicated by the need to interrogate a large genomic space and by many mutations leading to phenotypic minimum inhibitory concentrations close to the critical concentration.^6, 8–10^ Nevertheless, our understanding of genotypic–phenotypic correlation continues to improve,^6^ and genotypic techniques are a pragmatic pathway to drug susceptibility testing scale-up given the slow growth rate and biohazards of working with live *M. tuberculosis*. Our simplified solution to targeted NGS sample processing requires only the loading of reagent-treated sputum into a GeneXpert cartridge, producing as output an individual sequencing library. Library quantification can be eliminated by controlling the maximum number of amplicons for Illumina cluster generation by limiting our Illumina adapter-tailed primers. The microfluidic sample processing is similar to the existing globally utilized Xpert Ultra assay, with a 2-hour turnaround time, and will be relevant to detecting resistance to a new generation of oral regimens in high-burden settings. The automated in-cartridge sample processing and DNA isolation process reduces user variation, simplifies DNA extraction, and removes the multiple enzymatic reactions and need for costly kits involved in standard library preparation.^6^

There are some limitations to our current approach. First, in these proof-of-concept experiments, we have only targeted two genes. However, we sequenced their entire length, covering most clinically relevant BDQ resistance-conferring mutations. We plan to incorporate all the targets in the WHO resistance catalogue,^6^ meaning there is more template for the second-round PCR primers (that add the Illumina barcodes) in low bacillary load samples. This will reduce PCR artifacts while providing valuable information. Second, we process one individual sample per Xpert cartridge, maximizing flexibility but limiting the speed with which samples can be run simultaneously. Where high throughput is desirable, sputum could be alternatively processed on larger-module GeneXpert instruments that are already available in many high-burden laboratories. Generated libraries could be pooled along with any standard Illumina library, allowing for batching (even with other sample types) to reduce sequencing cost. Finally, PCR artifacts (primer-dimers) caused a sequence quality drop-off and sub-optimal alignment rate at the ends of the amplicons, though mutation calling across the entirety of both genes in all clinical samples was not affected. Such artifacts occur at higher rates in low bacterial load samples, but can be reduced by adding more targets or reducing primer concentrations.

In conclusion, we have developed a novel hemi-nested PCR technique contained entirely within a GeneXpert microfluidic cartridge for sample processing, cell lysis, DNA extraction and library construction. For the first time, this allows for sequencing of amplicons longer than 600 nucleotides on an Illumina instrument, uniting a well-established diagnostic system with a state-of-the-art sequencing platform. This technique dramatically simplifies conventional sequencing library preparation and could provide a streamlined workflow in routine laboratories for rapid individualized RR-TB treatment regimens.

## Supplementary Material


