# Supplementary material for: Cartridge-based sequencing for bedaquiline resistance detection from sputum
Source: IJTLD Open. 2024 Sep 1;1(9):422–4. doi: 10.5588/ijtldopen.24.0124 (PMC11409173; doi:10.5588/ijtldopen.24.0124)
Supplement: Supplementary file 1 [file ijtldopen24-0124_supplementarydata1.docx]

**Cartridge-based sequencing for bedaquiline resistance detection from sputum**

Jason D Limberis^1^, Roland J Nagel^2^, Dena Block^2^, Rebecca E Colman^3^, Alina Nalyvayko^4^, Zach Howard^4^, Scott Dewell^2^, Soumitesh Chakravorty^2^, John Z Metcalfe^1^

**Affiliations:** ^1^Division of Pulmonary and Critical Care Medicine, Zuckerberg San Francisco General Hospital and Trauma Centre, University of California, San Francisco, San Francisco, CA, USA; ^2^Cepheid Inc., Sunnyvale, California, USA; ^3^Division of Pulmonary, Critical Care, Sleep Medicine, and Physiology, University of San Diego, San Diego, California, USA. ^4^Division of Experimental Medicine, University of California, San Francisco, San Francisco, CA, USA.

**Running title:** Cartridge-based bedaquiline resistance seq

**Correspondence (and to whom reprint requests** should be addressed):

**John Z. Metcalfe, M.D., Ph.D., M.P.H.**

University of California, San Francisco

Division of Pulmonary and Critical Care Medicine

Zuckerberg San Francisco General Hospital and Trauma Center

University of California, San Francisco

CA 94110–0111, USA

Fax: (415) 695-1551

Email: [john.metcalfe@ucsf.edu](mailto:john.metcalfe@ucsf.edu)

**Word count:** 987

**Keywords:** Drug-resistant tuberculosis; bedaquiline; targeted next-generation sequencing

**Funding:** The NIH National Institute of Allergy and Infectious Diseases supported this work (R01AI177637 and U01AI152087).

**Supplementary Data*.***


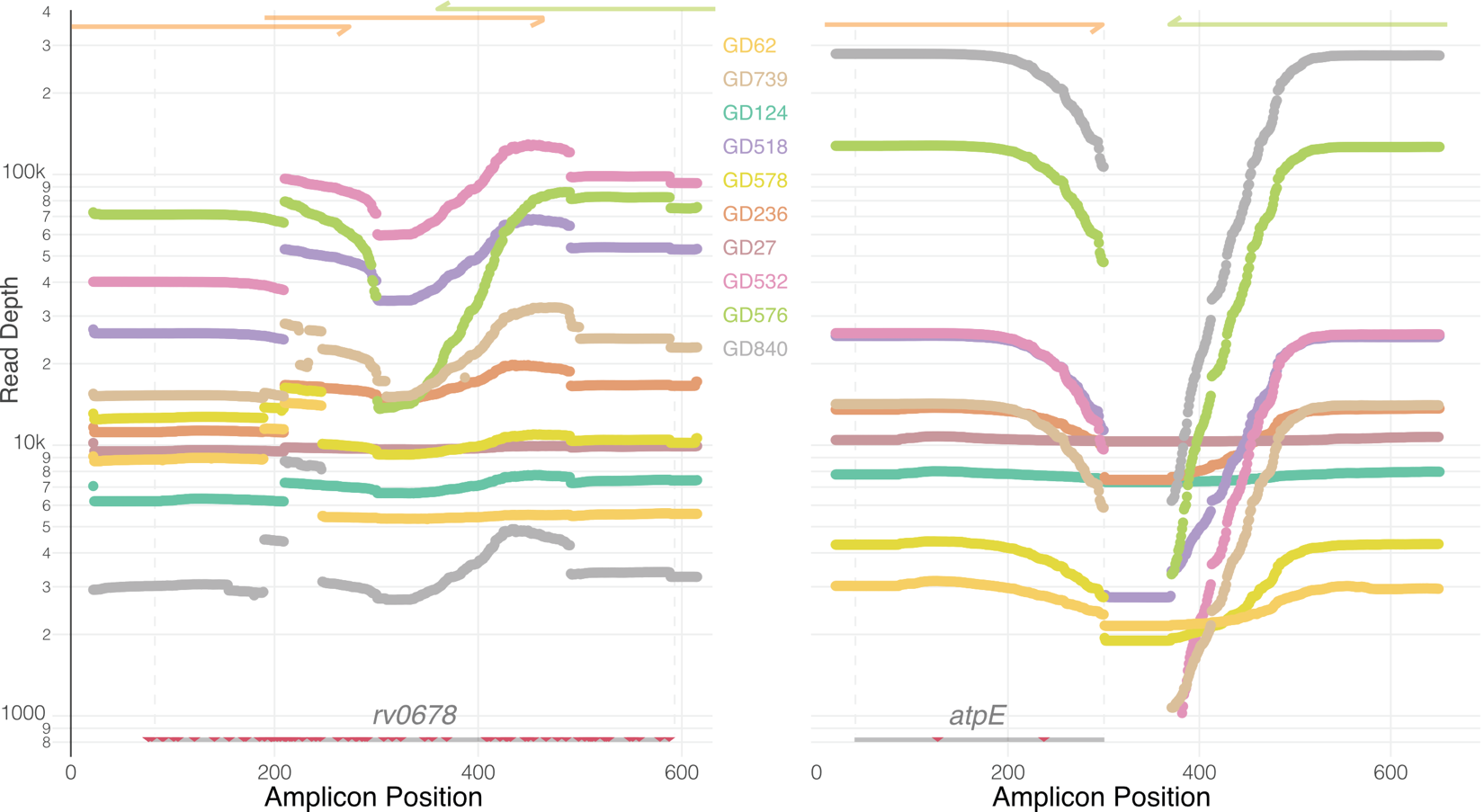


**Supplementary Figure 1. Successful sequencing of *rv0678* and its promoter region and *atpE* from clinical sputum samples with DNA extraction and library preparation performed entirely in an Xpert cartridge.** The plot shows the coverage (y-axis) at each position (x-axis) along the sequence amplicons. Coding regions for each gene are denoted by grey lines at the bottom of the figure, and red triangles note known bedaquiline resistance-conferring mutation locations^7,10^. The positions of forward primer amplicons are shown in orange at the top of each plot, with reverse primer amplicons in green. The drop-off in quality at the ends of the 300bp Illumina sequencing reads is expected (most visible in the *atpE* containing amplicon), as is the missing coverage in the *atpE* amplicon (however, the entirety of *atpE* is covered).

**Supplementary Table 1.** Primers used in this study.

| Gene | Name | Direction | Sequence |
| --- | --- | --- | --- |
| AtpE | atpE_stilPCRm2_v3_R1 | R | GACTGGAGTTCAGACGTGTGCTCTTCCGATCTGGACGCGTGCGTCCTCGATG |
| AtpE | atpE_stilPCRm2_v3_F1 | F | ACACTCTTTCCCTACACGACGCTCTTCCGATCTACCGGATGCTGGTAACGGCT |
| Rv0678 | Rv0678_stilPCR2_v5_F1 | R | ACACTCTTTCCCTACACGACGCTCTTCCGATCTAGTTCCAATCATCGCCCTCCGC |
| Rv0678 | Rv0678_stilPCR2_v5_F2 | F | ACACTCTTTCCCTACACGACGCTCTTCCGATCTTGACTCGGTTGGCGGGTCGA |
| Rv0678 | Rv0678_stilPCR2_v5_R | F | GACTGGAGTTCAGACGTGTGCTCTTCCGATCTGCCGTCTTGCTCGCCACCTC |
| AtpE | atpE_stilPCRm2_v3_R1 | R | GACTGGAGTTCAGACGTGTGCTCTTCCGATCTGGACGCGTGCGTCCTCGATG |
| AtpE | atpE_stilPCRm2_v3_F1 | F | ACACTCTTTCCCTACACGACGCTCTTCCGATCTACCGGATGCTGGTAACGGCT |
| UT | IlluminaAdapt_Full_F | F | AATGATACGGCGACCACCGAGATCTACAC[i5]ACACTCTTTCCCTACACGACGCTCTTCCGATCT |
| UT | IlluminaAdapt_Full_R | R | GATCGGAAGAGCACACGTCTGAACTCCAGTCAC[i7]ATCTCGTATGCCGTCTTCTGCTTG |
